# The heritability of body composition

**DOI:** 10.1186/s12887-021-02695-z

**Published:** 2021-05-08

**Authors:** Avivit Brener, Yarden Waksman, Talya Rosenfeld, Sigal Levy, Itai Peleg, Adi Raviv, Hagar Interator, Yael Lebenthal

**Affiliations:** 1 Pediatric Endocrinology and Diabetes Unit, Dana-Dwek Children’s Hospital, Tel Aviv Sourasky Medical Center, Tel Aviv, Israel; 2 Sackler Faculty of Medicine, Tel Aviv University, Tel Aviv, Israel; 3 The Nutrition & Dietetics Unit, Dana-Dwek Children’s Hospital, Tel Aviv Sourasky Medical Center, Tel Aviv, Israel; 4 Statistical Education Unit, The Academic College of Tel Aviv Yaffo, Tel Aviv, Israel

**Keywords:** Body composition, Bioimpedance, Children and adolescents, Heritability, Puberty

## Abstract

**Background:**

Physical growth during childhood and adolescence is influenced by both genetic and environmental factors. Heritability, the proportion of phenotypic variance explained by genetic factors, has been demonstrated for stature and weight status. The aim of this study was to explore the heritability of body composition.

**Methods:**

A real-life, observational study of the children and adolescents referred to the Endocrine Unit in a tertiary medical center. In January 2018, body composition by means of bioimpedance analysis (BIA) was implemented as part of the standard intake assessment of subjects referred for endocrine consultation. The clinic BIA database was searched for subjects with the term “observation of growth” as the sole reason for referral. BIA of 114 triads of healthy subjects aged 5–18 years and their parents were analyzed. The BIA report included the following data: fat mass, fat percentage, truncal fat percentage and muscle mass. Calculated variables included: appendicular skeletal muscle mass (ASMM = the sum of muscle mass of four limbs), muscle-to-fat ratio [MFR = ASMM (kg)/fat mass (kg)] and sarcopenic index [(SI = ASMM(kg)/height (meter)²]. Data collection from medical files included pubertal stage and home address for socioeconomic position grading.

**Results:**

There were sex differences in body composition parameters in both the prepubertal and pubertal subjects. The boys among the prepubertal subjects had a lower fat percentage on average than girls (*p* = 0.020). Among the adolescents, boys on average had lower fat percentage (*p* = 0.011), higher sarcopenic index (*p* = 0.021), and higher muscle-to-fat ratio (*p* < 0.001), than adolescent girls. Correlation analyses between body composition parameters of all participants revealed significant correlations in the sarcopenic index of prepubertal children and their parents (boys-fathers: r = 0.380, *p* = 0.050; boys-mothers: r = 0.435, *p* = 0.026; girls-fathers: r = 0.462, *p* = 0.012; girls-mothers: r = 0.365, *p* = 0.050) and adiposity indices (fat percentage, truncal fat percentage and muscle-to-fat ratio) of prepubertal boys and their mothers (r = 0.438, *p* = 0.025; r = 0.420, *p* = 0.033, and r = 0.478, *p* = 0.014, respectively). There were no associations between body composition parameters of adolescents and their parents. Socioeconomic position adversely affected fat percentage in adolescent girls and mothers.

**Conclusions:**

Heritable body composition traits were demonstrated in childhood but not in adolescence, suggesting that environmental influence has a more telling effect during teenage years.

## Introduction

Physical growth during childhood and adolescence is influenced by both genetic and environmental factors. Heritability, the proportion of phenotypic variance explained by genetic factors, has been studied for physical characteristics. Stature was shown as being highly heritable [1], but the extent of variation attributed to environmental influence is controversial [2]. This was demonstrated by studies of identical twins and families [3] and by genome-wide association studies (GWAS) [4, 5]. Genetic influence on weight status, as expressed by body mass index (BMI), has also been extensively studied, with multiple reports on heritability [6, 7]. BMI was demonstrated as a transmissible property shown during childhood [1] and extending into adulthood [8], with an increased degree of heritability in men compared to women [9] and in younger adults compared to the elderly [9].

In the pediatric population, BMI together with the application of sex- and age-specific cutoffs, is the most widely used method for categorizing weight status [10]. However, since BMI does not distinguish muscle from adipose tissue, it may underdiagnose subjects with abnormal body composition [11, 12]. Body composition parameters, such as adipose tissue, muscle mass, and the distribution of adiposity, provide a more comprehensive analysis of weight status. Increased body fat percentage and increased abdominal obesity beginning in childhood are strong predictors and key factors in the development of early-onset type 2 diabetes and metabolic syndrome [13]. A relative decreased muscle mass is also linked to metabolic derangements [14]. The heritability of these parameters, however, remains unclear.

In January 2018, our Pediatric Endocrine Unit implemented the analysis of body composition by means of bioimpedance analysis (BIA) as part of the standard intake assessment of subjects referred for endocrine consultation [15]. The BIA tool for assessing body composition was chosen since it is convenient, cost-effective, non-invasive and quick to perform, and provides a more nuanced glimpse into the subject’s weight status and overall health. Body composition assessment by means of BIA relies upon a calibration equation that uses a reference method, such as dual-energy X-ray absorptiometry, computed tomography or magnetic resonance imaging. BIA measurements have dramatically improved over recent years due to the development of sophisticated software that employs advanced algorithms. We collected BIA measurements of the children and adolescent as well as their parents, speculating that body composition parameters may be heritable. The aim of this study was to examine the interaction of body composition parameters between parents and their offspring to test that hypothesis.

## Methods

### Study design

This real-life, observational study of the children and adolescents referred to the Endocrine Unit in a tertiary medical center extended from January 2018 to July 2020. The clinic BIA database was searched for subjects with the term “observation of growth” as the sole reason for referral. The BIA data were linked to the subjects’ electronic medical records. Included in the study were healthy subjects aged 5 to 18 years with BMI z-scores between − 2.5 and 2.5 and the availability of BIA measurements for both biologic parents. Excluded were subjects with a medical history of prematurity, intrauterine growth retardation, born small or large for gestational age, and an underlying medical disorder or chronic medication administration affecting growth.

### Data collection

Data collected from the participants’ medical files at the time of BIA assessment included sex, age, anthropometric measurements, pubertal stage according to Marshall and Tanner [16, 17], and home address.

### Anthropometric measurements and pubertal assessment

The clinical evaluation of subjects and their parents included measurement of height, standing with bare feet by a commercial Harpenden stadiometer (Holtain Ltd., Crosswell, United Kingdom). The height measurements were performed to the nearest 0.1 centimeter, each measurement was repeated and the mean of the 2 measurements was recorded. Body weight and body composition were measured in light clothing by BIA. Body composition assessments were not performed in pregnant and lactating women. BMI was calculated as weight in kilograms divided by height in meters squared. The subjects’ height, weight, and BMI values were converted to sex- and age-specific standard deviation scores (z-scores) according to the CDC 2000 growth charts [18]. The parents’ height was recorded in absolute values and z-scores. The mid-parental height (MPHt) was calculated as follows: (paternal height [cm] + maternal height [cm] ± 13 cm)/2, and MPHt z-scores were calculated accordingly [19]. Delta height z-score represented the subject’s height compared to the potential genetic height, and it was calculated as the difference between the height z-score and the MPHt z-score.

Physical examinations with pubertal staging were routinely performed at clinic visits by pediatric endocrinologists at each BIA assessment. Prepubertal and pubertal stages were graded with Tanner scores for genital status in boys and for breast development in girls [16, 17]. Onset of puberty was defined as genitalia Tanner stage 2 with testicular volume > 3 mL in boys and appearance of breast buds in girls, with or without sexual hair. The subject was considered fully pubertal when pubertal signs corresponded to Tanner stage 5.

### Measurement of body composition

Body composition in subjects older than 5 years of age who were able to stand upright was measured by BIA (Tanita Body-Composition Analyzer, Tanita MC-780 MA and GMON Professional Software), which has been clinically verified to be accurate and reliable and to provide highly reproducible results [20, 21]. The BIA measures both whole body and segmental analysis (trunk, upper and lower limbs) of fat and muscle. The BIA measurements of subjects and parents were performed during the routine clinic visit, in the morning (from 8:00 AM until 13:00), preferably with the subject in a fasting state and not after strenuous physical activity. It required the subject to stand barefoot on the analyzer and grip the handles. The entire procedure took approximately 1 min per subject. The BIA report includes the following data: body weight (kilograms up to 270 kg in 0.1 kg increments), fat percentage (FATP of the whole body from 1.0 to 75.0 % in 0.1 % increments), truncal fat percentage (TFATP), fat mass (kilograms in 0.1 kg increments), fat-free mass (whole body in 0.1 kg increments), total body water percentage (TBW in 0.1 % increments), and basal metabolic rate (BMR expressed in kcal in 1 kcal increments).

Calculated variables included: appendicular skeletal muscle mass (ASMM = the sum of muscle mass of four limbs), muscle-to-fat ratio [MFR = ASMM (kg)/fat mass (kg)] and sarcopenic index [(SI = ASMM (kg)/height (meter)²].

### Socioeconomic position (SEP)

The SEP by home address was analyzed based on the Israel Central Bureau of Statistics’ Characterization and Classification of Statistical Areas within Municipalities and Local Councils by the Socio-Economic Level of the Population 2015 [22]. The neighborhoods or localities are divided into SEP clusters, with 1 representing the lowest and 10 representing the highest rating. The SEP index is an adjusted calculation of 14 variables that measure social and economic levels in the domains of demographics, education, standard of living, and employment (-2.797 to 2.590).

### Statistical analysis

The data were analyzed with the Statistical Package for the Social Sciences software version 26 (SPSS Inc., Chicago, IL, USA). All statistical tests were two-sided. The data are expressed as means ± standard deviations (SDs) for continuous variables and as counts and percentages for categorical variables. One-way analysis of variance (ANOVA) was used for comparing the means of continuous variables. The χ2 test was used to compare groups in categorical variables. Pearson correlations were applied to determine associations between body composition parameters of the subjects and their parents, as well as associations between the SEP scores and the body composition parameters. A *p* value of ≤ 0.05 was considered significant.

## Results

### The characteristics of the children and adolescents

One-hundred and fourteen pediatric subjects (58 boys, 51 %), mean age 10.3 ± 2.9 years, were included in the study. The clinical characteristics of the children and adolescents stratified by sex are presented in Table 1. The mean SEP cluster was 8.2 ± 1.1 (range 2 to 9) and the mean SEP index was 1.489 ± 0.597 (range − 0.907 to 2.438). The mean height z-score ± SD of subjects was − 0.29 ± 1.38, their MPHt z-score ± SD was − 0.04 ± 0.87 and their delta height z-score ± SD was − 0.25 ± 1.16. The subjects’ mean weight z-score ± SD was − 0.24 ± 1.48, with BMI z-scores of -0.03 ± 1.21, without significant differences in weight status (BMI z-scores) between the sexes. Fifty-five patients (48.3 %) were pre-pubertal Tanner stage 1, 43 (37.7 %) were within puberty Tanner stage 2–4, and 16 (14 %) were fully pubertal (Tanner stage 5), without significant differences in pubertal status distribution between boys and girls (*p* = 0.470).

**Table 1:** Sociodemographic and anthropometric parameters of 114 subjects stratified by sex

Parameter	Boys	Girls	*p*
Number	58	56	
Age, years	11.0 ± 2.9	9.7 ± 2.8	**0.016**
**Socioeconomic position**			
Socioeconomic position, cluster	8.2 ± 0.8	8.1 ± 1.3	0.376
Socioeconomic position, index	1.564 ± 0.548	1.411 ± 0.639	0.172
**Anthropometric parameters**			
Height, cm	140 ± 20	137 ± 18	0.450
Height, z-score	-0.68 ± 1.14	0.12 ± 1.50	**0.002**
Mid-parental height, z-score	-0.22 ± 0.69	0.15 ± 0.99	**0.021**
Delta height, z-score	-0.46 ± 1.20	-0.04 ± 1.09	**0.050**
Weight, kg	37.7 ± 18.3	36.0 ± 17.6	0.599
Weight, z-score	-0.57 ± 1.46	0.09 ± 1.44	**0.016**
Body mass index, z-score	-0.21 ± 1.26	0.15 ± 1.13	0.111
**Pubertal status**			
Tanner 1, n (%)	26 (44.8)	29 (51.8)	0.470
Tanner 2–4, n (%)	26 (44.8)	17 (30.4)	
Tanner 5, n (%)	6 (10.4)	10 (17.8)	
Data are presented as mean ± standard deviation unless otherwise specified. The socioeconomic position by home address was analyzed based on the Israel Central Bureau of Statistics’ Characterization and Classification of Statistical Areas within Municipalities and Local Councils by the Socio-Economic Level of the Population 2015. The SEP cluster classifies neighborhoods and localities into clusters, with 1 being the lowest rating and 10 the highest. The SEP index is an adjusted calculation of 14 variables that measure social and economic levels in four domains: demographics, education, standard of living, and employment. The mid-parental height was calculated as follows: (paternal height [cm] + maternal height [cm] ± 13 cm)/2 and presented as z-scores. Delta height z-score was calculated as the difference between the patient’s height z-score and the mid-parental height z-score. Bold values denote statistical significance at the *p* ≤ 0.05 level			

There were sex differences in body composition parameters in both the prepubertal and pubertal subjects. The body composition parameters stratified by sex and pubertal status are presented in Table 2. The boys among the prepubertal subjects had a lower FATP on average (*p* = 0.020), a higher TBW (*p* = 0.018), and higher BMR values (*p* = 0.005) than the girls. Among the adolescents, there were sex differences in body composition parameters, with adolescent boys on average having lower FATP (*p* = 0.011), higher TBW (*p* = 0.011), higher MFR (*p* < 0.001), higher SI (*p* = 0.021), and higher BMR values (*p* = 0.003) than adolescent girls. Body composition parameters differed significantly between prepubertal children and adolescents.

**Table 2:** Body composition parameters of subjects stratified by sex and pubertal status

	Prepubertal children			Adolescents				
**Parameter**	**Boys** ***n* = 26**	**Girls** ***n* = 29**	***p***^***1***^	**Boys** ***n* = 32**	**Girls** ***n* = 27**	***p***^***2***^	***p***^***3***^	***p***^***4***^
Age, years	8.5 ± 2.1	8.1 ± 1.5	0.483	13.0 ± 1.7	11.3 ± 2.8	**0.007**	**< 0.001**	**< 0.001**
Body mass index, z-score	-0.63 ± 1.23	-0.14 ± 1.26	0.145	0.14 ± 1.19	0.47 ± 0.89	0.247	**0.019**	**0.045**
Fat percentage	19.9 ± 5.6	23.3 ± 4.9	**0.020**	22.0 ± 8.6	27.3 ± 6.8	**0.011**	0.301	**0.015**
Truncal fat percentage	15.7 ± 5.9	17.5 ± 5.3	0.232	17.9 ± 8.7	21.5 ± 7.3	0.094	0.270	**0.023**
Fat free mass, kg	19.4 ± 5.9	19.9 ± 4.8	0.695	36.9 ± 11.1	32.6 ± 10.4	0.129	**< 0.001**	**< 0.001**
Appendicular skeletal muscle mass, kg	6.41 ± 3.09	7.04 ± 2.13	0.382	15.08 ± 5.57	12.53 ± 4.78	0.066	**< 0.001**	**< 0.001**
Total body water, percentage	58.6 ± 4.1	56.1 ± 3.6	**0.018**	57.2 ± 6.3	53.2 ± 5.0	**0.011**	**< 0.001**	**< 0.001**
Muscle-to-fat ratio	1.34 ± 0.41	1.19 ± 0.26	0.099	1.59 ± 0.53	1.05 ± 0.24	**< 0.001**	**0.050**	**0.045**
Sarcopenic index, kg/m^2^	3.97 ± 1.10	4.33 ± 0.71	0.149	6.20 ± 1.42	5.39 ± 1.13	**0.021**	**< 0.001**	**< 0.001**
Basal metabolic rate, kcal	1114.0 ± 135.6	1019.2 ± 104.5	**0.005**	1494.9 ± 254.5	1287.0 ± 254.5	**0.003**	**< 0.001**	**< 0.001**
Data are presented as mean ± standard deviation. Appendicular skeletal muscle mass (ASMM) = the sum of muscle mass of four limbs, muscle-to-fat ratio [MFR = ASMM (kg) /fat mass (kg)] and sarcopenic index [(SI = ASMM (kg) / height (meter)²]. *P*^*1*^ compares between prepubertal boys and girls; *p*^*2*^ compares between adolescent boys and girls; *p*^*3*^ compares between prepubertal and adolescent boys and *p*^*4*^ compares between prepubertal and adolescent girls. Bold values denote statistical significance at the *p* ≤ 0.05 level								

### The characteristics of the parents

The fathers were 3 years older on average than the mothers (46.6 ± 4.8 years and 43.7 ± 4.1 years, respectively, *p* < 0.001). The mean height of the parents was 176.7 ± 6.6 cm for fathers and 162.8 ± 7.7 cm for mothers. The mean BMI was 26.3 ± 3.7 and 23.3 ± 4.5, for the fathers and the mothers, respectively, *p* < 0.001. A comparative analysis between the parents of boys and girls (fathers of boys vs. girls and mothers of boys vs. girls) revealed no significant differences in age, height, and BMI.

### Correlation analyses between the subjects and their parents

**Fig. 1:**
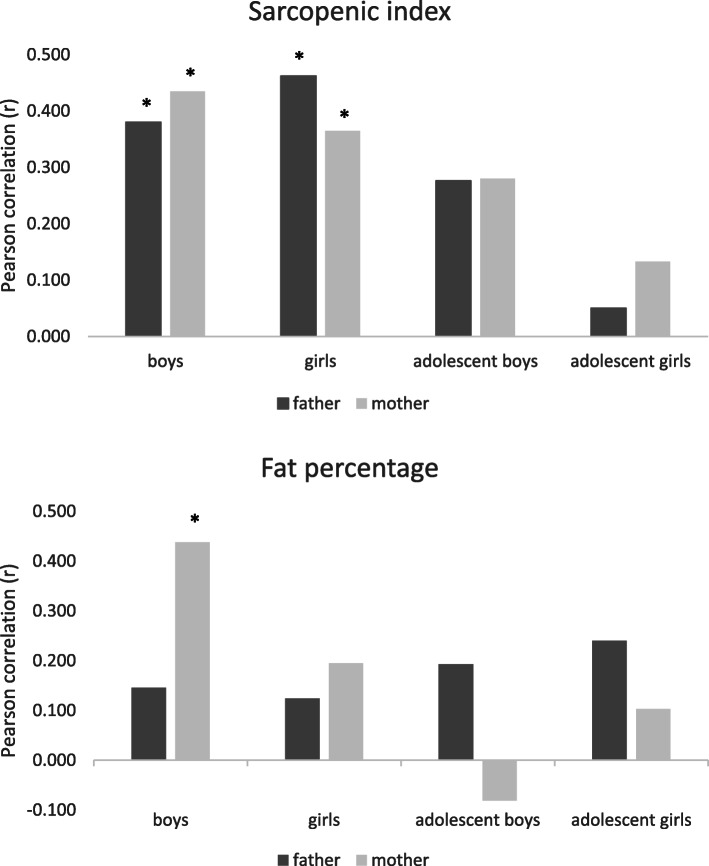
Histogram representation of Pearson correlation of body composition parameters between subjects (stratified by sex and pubertal status) and their fathers/mothers. Correlations analyses for sarcopenic index (Panel **a**) between prepubertal boys/girls and their fathers/mothers were significant (boys-fathers: r = 0.380, *p* = 0.050; boys-mothers: r = 0.435, *p* = 0.026; girls-fathers: r = 0.462, *p* = 0.012; girls-mothers: r = 0.365, *p* = 0.050). There were no significant correlations between adolescent boys/girls and their fathers/mothers. Correlation analyses for fat percentage (Panel **b**) between subjects and their fathers/mothers revealed a significant correlation only between prepubertal boys and their mothers (FATP: r = 0.438, *p* = 0.025). Asterisk indicates significance

The height z-scores of the subjects correlated significantly with the height z-scores of their fathers and mothers (r = 0.443, *p* < 0.001 and r = 0.483, *p* < 0.001, respectively). The BMI z-scores of the subjects were also correlated significantly with the BMI z-scores of their fathers and mothers (r = 0.228, *p* = 0.014 and r = 0.238, *p* = 0.011, respectively). Correlation analyses between body composition parameters of the subjects (stratified by sex and pubertal status) and their parents revealed significant correlations between FATP, TFATP and MFR values in prepubertal boys and their mothers (FATP: r = 0.438, *p* = 0.025; TFATP: r = 0.420, *p* = 0.033; MFR: r = 0.478, *p* = 0.014). Significant correlations were found in the SI values between prepubertal boys/girls and their fathers/mothers (boys-fathers: r = 0.380, *p* = 0.050; boys-mothers: r = 0.435, *p* = 0.026; girls-fathers: r = 0.462, *p* = 0.012; girls-mothers: r = 0.365, *p* = 0.050). Correlations between body composition parameters of adolescent boys and girls and their parents did not reach levels of significance. Figure 1 is a graphical depiction of the correlations of FATP and SI.

Correlation analyses between body composition parameters of the subjects (stratified by sex and pubertal status) and SEP scores (cluster and index) revealed significant negative correlations between FATP and SEP cluster/index for adolescent girls (r = -0.500, *p* = 0.008 and r = -0.435, *p* = 0.023) and their mothers (r = -0.208, *p* = 0.026 and r = -0.286, *p* = 0.002). No other significant correlations were found between SEP scores and body composition parameters.

## Discussion

The main finding of this investigation into the relationship between the body composition of healthy children and adolescents to their parents was a sex-dependent interaction between body composition parameters of the subjects and their parents. The muscle index (sarcopenic index) of prepubertal children was associated with that of both parents, while only the boys’ adiposity indices (total and truncal fat percentage and muscle-to-fat ratio) were associated with those of their mothers. Interestingly, there was no association between muscle and adiposity parameters of adolescents and those of their parents, pointing towards environmental influences that prevail over the genetic influences seen in prepubertal children.

In line with previous reports [1–4, 23], we found a strong association between the current height status of the subjects and their genetic height. The weight status of the subjects in our study, as expressed by the BMI z-score, was also observed to be a heritable trait, but to a lesser degree, suggesting a more diverse phenotypic variation [5, 6]. However, BMI is a poor proxy for adiposity and it does not distinguish muscle from adipose tissue, nor does it describe the distribution of adipose tissue. Further study that focuses on the heritability of body composition may provide insight into metabolic and cardiovascular health outcomes.

The strong interactions between prepubertal children and their parents demonstrated in this study were seen in the parameter of height-adjusted muscle mass (sarcopenic index). Variation in skeletal muscle traits among individuals can be attributed to genetic factors, environmental factors, or some interaction of both [24]. The influence of environmental factors, such as physical activity and diet, have been broadly investigated. More recent studies have begun to identify the specific genetic influences on skeletal muscle traits that may explain the inter-individual trait variability. Studies on muscle strength have reported distinct heritable patterns, with an increasing role of the environmental factors with age [25]. Family studies have estimated that heritability of the muscle mass indices ranged from 55 to 80 % [26, 27]. Recent GWAS studies have identified novel loci which determine lean body mass [28], thus revealing the genetic basis behind the clinical inspections. Environmental influences with time may disconnect the interaction between muscle indices of adolescents and their parents.

Differences in body fat distribution between the sexes emerge in childhood, become more apparent during puberty [11], and change throughout adulthood. Sexual dimorphism in human body composition is evident from fetal life, but it becomes more pronounced during puberty, primarily due to the action of sex steroid hormones [29]. At birth, males have a similar fat mass as females, but they are longer and have greater lean mass [30]. Adult males have greater muscle mass [31], larger and stronger bones [32], and reduced limb fat, with a similar degree of central abdominal fat, while females tend to have a more peripheral distribution of fat [33]. This physiologic course leading to pubertal differences in body habitus was also demonstrated in our current study. Environmental exposures with time may affect both muscle and fat tissue, thus leading the adolescent on a path of body composition that diverges from the parental pattern.

Body weight and adiposity are also influenced by the balance between energy intake and energy expenditure. Given the same BMI, adipose tissue proportion and distribution vary between the sexes: the feminine fat distribution is more peripheral while the masculine is more central [33]. These differences play a pivotal role in the development of insulin resistance and obesity-related complications since the accumulation of visceral fat is strongly linked to increased metabolic and cardiovascular risks [34]. It is well-established that the inheritance of adiposity is polygenic [35], and polymorphism in the *MC4R* gene was identified as one of the target genes responsible for adiposity determination [36]. Nevertheless, the phenotypic heritability of the genotypic polymorphism has not yet been clearly described, suggesting that multiple factors are involved. In animal models, differences in body composition were associated with specific mitochondrial DNA haplotypes, suggesting a strong genetic component inherited through the maternal lineage [37]. Mitochondrial inheritance may provide at least part of the explanation for the interaction that we found between the fat percentage and the truncal fat percentage of prepubertal boys and their mothers, without a similar interaction between fathers and their boys.

The heritability of body composition parameters may provide pathophysiologic explanations for the well-known heritability of metabolic and cardiovascular morbidity. Our findings suggest that body composition has heritable traits which differ between the sexes. This dimorphic behavior may result from the differences between the sexes in expression of the genes related to body composition. A meta-analysis of GWAS and Metabochip data in 224,000 individuals identified strong sexual dimorphism in the genetic regulation of fat distribution traits [38], a characteristic not observed for overall obesity as assessed by BMI [8].

The link between body composition of parent and child may be interpreted as the result of sharing the same socioeconomic circumstances. However, correlations between SEP scores and body composition parameters were found only in the parameter of fat percentage in adolescent girls and their mothers. SEP is a well-known determinant of health conditions, with a predominantly inverse relationship with obesity as measured by anthropometric measures [39, 40], and this relationship is stronger in women [39]. Social inequalities may contribute to an adverse fat distribution and decreased muscle mass which are linked to worrisome health outcomes, such as cardiovascular disease, diabetes, and poor physical capability [39]. There are abundant reports that link SEP and BMI, but studies on the relationship between SEP and body composition in childhood and adulthood are limited [41]. Our current results add to the cumulative data in this field.

We acknowledge limitations to this study that bear mention. One is the relatively small sample size and the cross-sectional design that precluded the provision of longitudinal information on the clinical course affecting body composition acquisition. Another limitation is the lack of questionnaires for evaluating nutritional and physical activity habits. In addition, due to the absence of consensus on a quantitative definition of MFR and SI for sex, age, and pubertal stage, we could not establish preferable and unpreferable cutoffs [42]. Nevertheless, comparing the BIA of pediatric subjects with both of their parents enabled us to describe the interaction between the body composition parameters of both groups. Of note, our cohort is comprised of a relatively homogeneous population belonging to the middle to high socioeconomic position. Another strength is in the uniformity of anthropometric and BIA measurements obtained by the same trained medical personnel in the same manner.

In summary, our findings suggest that the apple does not fall far from the tree. Both muscle and adiposity parameters demonstrated heritable traits dependent upon sex (child and parent) and the pubertal status of the child. Heritable body composition traits were demonstrated in childhood but not in adolescence, suggesting an environmental influence that takes a more obvious effect during teenage years. Since the subjects were not diagnosed as having an endocrine disorder, our findings are transferable to other pediatric populations as well. Further research is warranted to delineate the genotypic-phenotypic course of body composition acquisition and its implications on metabolic health.

## Data Availability

The datasets generated and analyzed during the current study are available from the corresponding author on reasonable request.

## Abbreviations

ASMM: Appendicular skeletal muscle mass
BIA: Bioimpedance analysis
BMI: Body mass index
FATP: Fat percentage
TFATP: Truncal fat percentage
MFR: Muscle-to-fat ratio
MPHt: Mid-parental height
SEP: Socioeconomic position
SI: Sarcopenic index
